# Supplementary material for: HIVprotI: an integrated web based platform for prediction and design of HIV proteins inhibitors
Source: J Cheminform. 2018 Mar 9;10:12. doi: 10.1186/s13321-018-0266-y (PMC5845081; doi:10.1186/s13321-018-0266-y)
Supplement: Supplementary file 2 — Additional file 2. Source code of HIVProtI web server. [file 13321_2018_266_MOESM2_ESM.zip › HIVprotI_Source-code/hivprotI/weka-3-6-6/doc/weka/classifiers/CheckClassifier.html]

CheckClassifier


---


|  |  |  |
| --- | --- | --- |
| **Overview** \| **Package** \| **Class** \| **Tree** \| **Deprecated** \| **Index** \| **Help** \| **Weka's home** | |  |
| **PREV CLASS**   **NEXT CLASS** | **FRAMES**    **NO FRAMES**     **All Classes** |  |
| SUMMARY: NESTED \| FIELD \| CONSTR \| METHOD | DETAIL: FIELD \| CONSTR \| METHOD |  |


---


## weka.classifiers Class CheckClassifier

```
java.lang.Object
  weka.core.Check
      weka.core.CheckScheme
          weka.classifiers.CheckClassifier
```

**All Implemented Interfaces:**: OptionHandler, RevisionHandler

---

``` public class CheckClassifier extends CheckScheme ```

Class for examining the capabilities and finding problems with
classifiers. If you implement a classifier using the WEKA.libraries,
you should run the checks on it to ensure robustness and correct
operation. Passing all the tests of this object does not mean
bugs in the classifier don't exist, but this will help find some
common ones.

Typical usage:

`java weka.classifiers.CheckClassifier -W classifier_name
classifier_options` 

CheckClassifier reports on the following:

- Classifier abilities
  - Possible command line options to the classifier
  - Whether the classifier can predict nominal, numeric, string,
    date or relational class attributes. Warnings will be displayed if
    performance is worse than ZeroR
  - Whether the classifier can be trained incrementally
  - Whether the classifier can handle numeric predictor attributes
  - Whether the classifier can handle nominal predictor attributes
  - Whether the classifier can handle string predictor attributes
  - Whether the classifier can handle date predictor attributes
  - Whether the classifier can handle relational predictor attributes
  - Whether the classifier can handle multi-instance data
  - Whether the classifier can handle missing predictor values
  - Whether the classifier can handle missing class values
  - Whether a nominal classifier only handles 2 class problems
  - Whether the classifier can handle instance weights
- Correct functioning
  - Correct initialisation during buildClassifier (i.e. no result
    changes when buildClassifier called repeatedly)
  - Whether incremental training produces the same results
    as during non-incremental training (which may or may not
    be OK)
  - Whether the classifier alters the data pased to it
    (number of instances, instance order, instance weights, etc)
  - Whether the toString() method works correctly before the
    classifier has been built.
- Degenerate cases
  - building classifier with zero training instances
  - all but one predictor attribute values missing
  - all predictor attribute values missing
  - all but one class values missing
  - all class values missing

Running CheckClassifier with the debug option set will output the
training and test datasets for any failed tests.

The `weka.classifiers.AbstractClassifierTest` uses this
class to test all the classifiers. Any changes here, have to be
checked in that abstract test class, too.


Valid options are:

```
 -D
  Turn on debugging output.
```

```
 -S
  Silent mode - prints nothing to stdout.
```

```
 -N <num>
  The number of instances in the datasets (default 20).
```

```
 -nominal <num>
  The number of nominal attributes (default 2).
```

```
 -nominal-values <num>
  The number of values for nominal attributes (default 1).
```

```
 -numeric <num>
  The number of numeric attributes (default 1).
```

```
 -string <num>
  The number of string attributes (default 1).
```

```
 -date <num>
  The number of date attributes (default 1).
```

```
 -relational <num>
  The number of relational attributes (default 1).
```

```
 -num-instances-relational <num>
  The number of instances in relational/bag attributes (default 10).
```

```
 -words <comma-separated-list>
  The words to use in string attributes.
```

```
 -word-separators <chars>
  The word separators to use in string attributes.
```

```
 -W
  Full name of the classifier analysed.
  eg: weka.classifiers.bayes.NaiveBayes
  (default weka.classifiers.rules.ZeroR)
```

```
 Options specific to classifier weka.classifiers.rules.ZeroR:
```

```
 -D
  If set, classifier is run in debug mode and
  may output additional info to the console
```

Options after -- are passed to the designated classifier.

**Version:**
:   $Revision: 1.33 $

**Author:**
:   Len Trigg (trigg@cs.waikato.ac.nz), FracPete (fracpete at waikato dot ac dot nz)

**See Also:**: `TestInstances`

---

**Nested Class Summary**

**Nested classes/interfaces inherited from class weka.core.CheckScheme**

`CheckScheme.PostProcessor`


**Constructor Summary**

`CheckClassifier()`


| **Method Summary** | |
| --- | --- |
| `void` | `doTests()`             Begin the tests, reporting results to System.out |
| `Classifier` | `getClassifier()`             Get the classifier used as the classifier |
| `java.lang.String[]` | `getOptions()`             Gets the current settings of the CheckClassifier. |
| `java.lang.String` | `getRevision()`             Returns the revision string. |
| `java.util.Enumeration` | `listOptions()`             Returns an enumeration describing the available options. |
| `static void` | `main(java.lang.String[] args)`             Test method for this class |
| `void` | `setClassifier(Classifier newClassifier)`             Set the classifier for boosting. |
| `void` | `setOptions(java.lang.String[] options)`             Parses a given list of options. |

**Methods inherited from class weka.core.CheckScheme**

`attributeTypeToString, getNumDate, getNumInstances, getNumInstancesRelational, getNumNominal, getNumNumeric, getNumRelational, getNumString, getPostProcessor, getWords, getWordSeparators, hasClasspathProblems, setNumDate, setNumInstances, setNumInstancesRelational, setNumNominal, setNumNumeric, setNumRelational, setNumString, setPostProcessor, setWords, setWordSeparators`

**Methods inherited from class weka.core.Check**

`getDebug, getSilent, setDebug, setSilent`

**Methods inherited from class java.lang.Object**

`equals, getClass, hashCode, notify, notifyAll, toString, wait, wait, wait`

**Constructor Detail**

### CheckClassifier

```
public CheckClassifier()
```


**Method Detail**

### listOptions

```
public java.util.Enumeration listOptions()
```

:   Returns an enumeration describing the available options.

    :   **Specified by:**: `listOptions` in interface `OptionHandler` **Overrides:**: `listOptions` in class `CheckScheme`
    :   **Returns:**: an enumeration of all the available options.

---


### setOptions

```
public void setOptions(java.lang.String[] options)
                throws java.lang.Exception
```

:   Parses a given list of options.
    Valid options are:

    ```
     -D
      Turn on debugging output.
    ```

    ```
     -S
      Silent mode - prints nothing to stdout.
    ```

    ```
     -N <num>
      The number of instances in the datasets (default 20).
    ```

    ```
     -nominal <num>
      The number of nominal attributes (default 2).
    ```

    ```
     -nominal-values <num>
      The number of values for nominal attributes (default 1).
    ```

    ```
     -numeric <num>
      The number of numeric attributes (default 1).
    ```

    ```
     -string <num>
      The number of string attributes (default 1).
    ```

    ```
     -date <num>
      The number of date attributes (default 1).
    ```

    ```
     -relational <num>
      The number of relational attributes (default 1).
    ```

    ```
     -num-instances-relational <num>
      The number of instances in relational/bag attributes (default 10).
    ```

    ```
     -words <comma-separated-list>
      The words to use in string attributes.
    ```

    ```
     -word-separators <chars>
      The word separators to use in string attributes.
    ```

    ```
     -W
      Full name of the classifier analysed.
      eg: weka.classifiers.bayes.NaiveBayes
      (default weka.classifiers.rules.ZeroR)
    ```

    ```
     Options specific to classifier weka.classifiers.rules.ZeroR:
    ```

    ```
     -D
      If set, classifier is run in debug mode and
      may output additional info to the console
    ```

    :   **Specified by:**: `setOptions` in interface `OptionHandler` **Overrides:**: `setOptions` in class `CheckScheme`
    :   **Parameters:**: `options` - the list of options as an array of strings **Throws:**: `java.lang.Exception` - if an option is not supported

---


### getOptions

```
public java.lang.String[] getOptions()
```

:   Gets the current settings of the CheckClassifier.

    :   **Specified by:**: `getOptions` in interface `OptionHandler` **Overrides:**: `getOptions` in class `CheckScheme`
    :   **Returns:**: an array of strings suitable for passing to setOptions

---


### doTests

```
public void doTests()
```

:   Begin the tests, reporting results to System.out

    :   **Specified by:**: `doTests` in class `CheckScheme`

---


### setClassifier

```
public void setClassifier(Classifier newClassifier)
```

:   Set the classifier for boosting.

    :   **Parameters:**: `newClassifier` - the Classifier to use.

---


### getClassifier

```
public Classifier getClassifier()
```

:   Get the classifier used as the classifier

    :   **Returns:**: the classifier used as the classifier

---


### getRevision

```
public java.lang.String getRevision()
```

:   Returns the revision string.

    :   **Returns:**: the revision

---


### main

```
public static void main(java.lang.String[] args)
```

:   Test method for this class

    :   **Parameters:**: `args` - the commandline parameters


---


|  |  |  |
| --- | --- | --- |
| **Overview** \| **Package** \| **Class** \| **Tree** \| **Deprecated** \| **Index** \| **Help** \| **Weka's home** | |  |
| **PREV CLASS**   **NEXT CLASS** | **FRAMES**    **NO FRAMES**     **All Classes** |  |
| SUMMARY: NESTED \| FIELD \| CONSTR \| METHOD | DETAIL: FIELD \| CONSTR \| METHOD |  |


---
